# How to Isolate a Plant's Hypomethylome in One Shot

**DOI:** 10.1155/2015/570568

**Published:** 2015-09-03

**Authors:** Elisabeth Wischnitzki, Eva Maria Sehr, Karin Hansel-Hohl, Maria Berenyi, Kornel Burg, Silvia Fluch

**Affiliations:** AIT Austrian Institute of Technology GmbH, Konrad-Lorenzstreet 24, 3430 Tulln, Austria

## Abstract

Genome assembly remains a challenge for large and/or complex plant genomes due to their abundant repetitive regions resulting in studies focusing on gene space instead of the whole genome. Thus, DNA enrichment strategies facilitate the assembly by increasing the coverage and simultaneously reducing the complexity of the whole genome. In this paper we provide an easy, fast, and cost-effective variant of MRE-seq to obtain a plant's hypomethylome by an optimized methyl filtration protocol followed by next generation sequencing. The method is demonstrated on three plant species with knowingly large and/or complex (polyploid) genomes:* Oryza sativa*,* Picea abies*, and* Crocus sativus*. The identified hypomethylomes show clear enrichment for genes and their flanking regions and clear reduction of transposable elements. Additionally, genomic sequences around genes are captured including regulatory elements in introns and up- and downstream flanks. High similarity of the results obtained by a* de novo* assembly approach with a reference based mapping in rice supports the applicability for studying and understanding the genomes of nonmodel organisms. Hence we show the high potential of MRE-seq in a wide range of scenarios for the direct analysis of methylation differences, for example, between ecotypes, individuals, within or across species harbouring large, and complex genomes.

## 1. Introduction

Chemical modifications of DNA and histones, known as epigenetic marks, regulate the access to the genetic information encoded in the DNA of eukaryotic cells. Thereby, epigenetic modifications can inheritably coordinate gene expression without changing the underlying DNA sequence. As such, epigenetic regulation is an additional layer in the genetic information of a cell influencing a plethora of biological processes [1, 2]. In plants, the most common mark of DNA methylation is 5-methylcytosine (5-mC) [3]. The cytosine can be methylated at CG, CHG, and CHH sites, where H represents nonguanine residues. Cytosine methylation is nonrandomly distributed in plants and is found primarily in repetitive regions of the genome that are enriched in transposable elements (TEs), centromeric repeats, or silent rDNA repeats. When DNA methylation occurs in promoter regions and within the gene space it is associated with differential gene expression [4, 5].

Based on whole genome DNA methylation analyses it is now widely accepted that methylation marks in plants fluctuate according to the cell, tissue, and organ in the vegetative and reproductive phases of a plant's life cycle [6, 7]. This epigenetic variation is of utmost importance not only during plant development but also in the response to environmental conditions. Most notably, cytosine methylation patterns acquired in response to abiotic or biotic stress are often inherited over one to several subsequent generations. Thereby, the epigenetic system reversibly stores information over time functioning as a “molecular memory.” This transgenerational inheritance of DNA methylation can in some cases lead to novel epialleles and phenotypes within populations and thereby mediates phenotypic plasticity [8].

Thus, epigenetic profiling is an increasingly popular strategy for understanding the genetic and environmental interactions behind many biological processes. Therefore, robust, cost-effective, and scalable assays are needed for studying epigenetic variation in diverse contexts. Over the past years numerous methods have been developed to study a plant's methylome (the methylated part of the genome) and hypomethylome (the nonmethylated part of the genome), whereby each method is accompanied by its strengths and limitations (reviewed in [9, 10]). Nowadays, sequencing-based methods especially present a unique opportunity to achieve comprehensive methylome or hypomethylome coverage.

The scientific goal to focus the sequencing efforts led to strategies to enrich either methylated or nonmethylated DNA regions. Immunoprecipitation followed by sequencing (MeDIP-seq) is used to obtain the methylated parts of genomes [11]. Due to the relatively low cost for acquiring genome-wide data, MeDIP-seq is very attractive and has recently been applied to complex plant genomes, such as poplar [12], maize [13], and rice [14]. One the contrary, to enrich the nonmethylated part of a genome (the hypomethylome), methylation-sensitive restriction enzymes have been used. Based on the fact that the gene body in plants is showing rather low methylation levels (hypomethylated) and that, in contrast, cytosine methylation is found predominantly in repetitive elements (e.g., transposable elements) [4], methylation-sensitive enzyme-based genome digests creating reduced representation library allow enriching gene related sequences [15, 16]. A widely applied variation of this methyl filtration (MF) approach is using the enzyme McrBC followed by cloning steps [17, 18]. The combination of MF with subsequent next generation sequencing (NGS) is termed MRE-seq (methylation-sensitive restriction enzyme-seq). This method has so far been predominantly applied in mammalian tissue for analysing methylation differences [19–21]. Although an enhanced MF method has been described in 2009 for plants [22], most of the recent studies in plants still study the hypomethylome through the McrBC-based MF [23–25], MSAP (methylation-sensitive amplified polymorphism [26, 27]), RLGS (Restriction Landmark Genome Scanning [28]), or methylation-sensitive Southern blotting [29].

Due to some limitations in MF techniques (reviewed by [9]), there is still potential to improve the MRE-seq in order to allow a wider application of the technique for the direct analysis of methylation differences between ecotypes and the role of epigenetics as a source of variation contributing to fitness and natural selection especially with regard to nonmodel organisms.

With the present study performed on the model organism rice (*Oryza sativa*) we demonstrate that with an improved MRE-seq method, the hypomethylome and thus the gene space of a plant can be easily accessed by the use of methylation-sensitive restriction enzymes followed by next generation sequencing. Using different bioinformatics approaches we show that performing* de novo* assembly with the MF sequences allows the reconstruction of a large proportion of the gene space including promoters without prior knowledge of the whole genome. Furthermore we confirm our results in small scale studies in the large genome of Norway spruce (*Picea abies*) and the triploid saffron crocus (*Crocus sativus*) genome.

Our method provides an easy tool for killing two birds with one stone: (1) the reduced representation library enriched for gene space can serve as cost-effective tool for analysing a plant's gene space depleted of repetitive elements comprising over 50–80% of the genome [30]; (2) with this representation of the hypomethylome, an easy comparative analysis of epigenetic variation among genotypes or tissues can be performed at an affordable price, even in a larger set of samples.

## 2. Material and Methods

### 2.1. Plant Material

Genomic DNA was prepared from leaves of the* Oryza sativa* ssp.* indica* variety SHZ-2A (seeds are kindly provided by R. Mauleon, IRRI International Rice Research Institute, Los Banos, Philippines), from a pool of stigmata of the* Crocus sativus* L. accession “LaMancha” (material kindly provided by O. Santana-Méridas, Servicios Periféricos de Agricultura, Centro Agrario de Albaladejito, Cuenca, Spain), and from needles of* Picea abies* (L.) H. Karst. (twigs kindly provided by S. Schüler, Department of Forest Genetics, Austrian Research Centre for Forests, Vienna, Austria) using DNeasy Plant mini kit (Qiagen) following the manufacturer's instructions.

### 2.2. Methyl Filtration with Size Selection through PCR

#### 2.2.1. Enzyme Selection

In order to improve the MF enrichment towards a higher coverage of the gene space and to adjust the previously reported technique [22] towards NGS, the enrichment potency of five different methylation-sensitive enzymes (AciI, HpaII, and Bsh1236I sensitive to CpG methylation, and MspI and PspGI sensitive to CpH/WpG methylation) was evaluated in a first step using rice as the model of choice by following the steps described below.

#### 2.2.2. Digestion and Ligation

Digestion of the genomic DNA and ligation of the adapters was performed simultaneously in a single reaction for each enzyme separately. 300 ng of genomic DNA and 4 *μ*L 10 mM of the preannealed adaptors PmeI_CGWA (5′-GCACGACTGTTTAAA-3′) and PmeI_CGB (5′-CGTTTAAACAGTCGT-3′, 5′ phosphorylated) were mixed in 50 *μ*L reaction volume supplemented either with AciI, Bsh1236I, HpaII, MspI, or PspGI (40 U, NEB) each in the corresponding NEB buffer. During the enzymatic digestion process, the cut DNA fragments were simultaneously ligated to the double stranded adaptors with 2 *μ*L T4 ligase (Thermo Scientific) and 2 mM ATP being present in the same reaction mix. After overnight incubation at 37°C, the reaction was stopped by heat inactivation at 65°C for 20 minutes and diluted 1 : 1 with water. Samples were extracted with phenol-chloroform followed by chloroform before precipitation with EtOH. Samples were dissolved in 100 *μ*L 0.5x NEB4 buffer.

#### 2.2.3. Amplification of the Adaptor Ligated DNA

For Illumina sequencing, fragments were attained by PCR amplification of the restriction digested and adaptor ligated genomic DNA samples. 1 *μ*L of digested and adaptor ligated DNA and 6 *μ*L of 10 *μ*M amplification primer PmeI_CG17 (5′-CACGACTGTTTAAACGG-3′) were used in a 50 *μ*L PCR reaction containing 2.5 U HotStart Polymerase (Qiagen), 1 *μ*L 25 mM MgCl_2_, and 1 *μ*L 20 *μ*M dNTPs. The PCR yielded 200–800 bp fragments under the following cycling conditions: 95°C for 15 minutes; 30 times 95°C/30 sec, 55°C/40 sec, 72°C/50 sec; 72°C for 5 minutes. The PCR reactions were precipitated in EtOH and DNA dissolved in 100 *μ*L 5 mM Tris buffer (pH 8.0). Eight parallel reactions were performed for each restriction enzyme setup in order to collect sufficient amount of DNA for subsequent sequencing.

#### 2.2.4. Removal of the Adaptor Sequences

To increase the length of the usable sequence information, the majority of the adaptor sequence was removed by PmeI digestion, the rare cutter site (GTTTAAAC) included in the adaptor sequence. 20 *μ*g of the PCR amplifications were digested with PmeI (NEB) in NEB4 buffer and supplemented with 100 ng/*μ*L BSA in two steps. First digestion was performed in a 200 *μ*L reaction volume, containing 200 U PmeI enzyme on 37°C for 2 hours followed by a subsequent volume increase to 250 *μ*L including additional 50 U PmeI and incubated for additional 2 hours. Finally the reaction was stopped at 65°C for 20 minutes.

### 2.3. Sequencing

The rice and Norway spruce fragments have been prepared as amplicon libraries and next generation sequencing was performed on Illumina's HiSeq 2000 using 100 bp paired end technology. The individual libraries (5 libraries of rice, each treated with one of the above mentioned enzymes, and 5 libraries of N. spruce, 4 treated with HpaII, and a whole genome snapshot library as control) were barcoded and sequenced together in a single lane. Library preparation and sequencing was done by GATC Biotech AG. The sequencing of the saffron crocus fragments was performed on an Illumina MiSeq machine (300 bp paired end reads). The library was prepared and barcoded using the TruSeq DNA PCR-Free LT Sample Preparation Kit has been quantified using the KAPA Library Quantification Kit on a standard qRT-PCR machine, and the quality has been checked on the Agilent Bioanalyzer using the Agilent High Sensitivity DNA kit. All kits have been applied according to the manufacturer's protocols. The sample was sequenced using the MiSeq Reagent Kit v3 according to manufacturer's protocols (Illumina Inc.) together with one other sample.

### 2.4. Sequencing Data Processing

All sequence reads were cleaned in order to guarantee high quality data by removing adaptor fragments, low quality regions (Q30), and short sequences (<50 bp; <100 bp for saffron crocus MiSeq data) from the datasets using* in house* developed Perl scripts. Then sequence reads were analysed for their origin from potential repetitive elements (REdat version 9.3 [31]), ribosomal data (*in house* reference database based on ribosomal data from NCBI and unpublished* in house* data), and chloroplast or mitochondrion DNA (*Oryza sativa* ssp.* japonica* release 7 [32],* Picea abies* release 1.0 [33]). Due to the lacking genome sequence of saffron crocus the rice genomic data was used as reference for this step. The TE-related reads were included in the further analysis to avoid an artificial bias against TEs.

All coverage calculations were performed by dividing the sum of base pairs of the respective dataset by the size of the studied sequences. The simulation to estimate the minimal coverage necessary to identify the hypomethylome of the whole genome was performed on the rice dataset by randomly selecting reads from the combined dataset with subsequent mapping to the genome sequence (*Oryza sativa* ssp.* japonica* release 7 [32]) using bowtie2 with default settings [34]. The identified regions were compared to the genomic area covered with the complete combined dataset using bedtools (version: 2.17.0; [35]) and the resulting overlap was calculated.

### 2.5. Additional Analysis of Genome Sequences

The separation of gene models into genes and TEs, the identification of the 1.000 bp up- and downstream flanking regions as well as exon and intron regions in the analysed genomes, is based on the annotation of genome release 7 for rice and genome release 1.0 for Norway spruce.

Frequencies of AciI and HpaII restriction sites in the genomic sequence of rice [32],* Arabidopsis* (TAIR10; [36]), poplar (JGI 2.0; [37]), grapevine (Genoscope_v1; [38]), Norway spruce (v1.0; [33]), maize (5b.60; [39]), sorghum (JGI 1.4; [40]), and* Brachypodium* (MIPS 1.2; [41]) were calculated using* in house* developed Perl scripts. The information of AciI and HpaII frequencies in* Homo sapiens* was taken from http://tools.neb.com/~posfai/TheoFrag/TheoreticalDigest.human.html.

Known regulatory elements from publicly available resources (JASPAR, Agris, AthaMap, Transfac, PLACE [42–46]) were filtered for degenerated sites and a minimal length of eight nucleotides to minimize the probability for the detection of nonfunctional patterns due to random occurrences within the sequences. The remaining elements were located in the rice genome and assigned to the identified regions using* in house* developed Perl scripts.

### 2.6. Reference Based and* De Novo* Assembly

The reference guided assembly was performed by assigning all high quality read sequences to the genome sequences of rice [32] or Norway spruce [33], respectively, using bowtie2 with default settings [34] resulting in regions representing the hypomethylated fragments. This was performed for each of the datasets separately. In order to guarantee that the regions used during downstream analyses do not represent false positives due to problems during the mapping, sequencing errors or technical problems during wet lab processes, only genomic regions were retained comprising at least five reads.

The* de novo* assembly for each enzyme and the combined dataset for rice and the dataset of saffron crocus was performed using Trinity [47] with a minimal contig length of 100 bp. The resulting contigs were evaluated by mapping the high quality reads used for the assembly to the assembled contig sequences using bowtie2 [34] and only contigs consisting of at least five reads were retained, similar to the reference based assembly. The contigs assembled for the rice datasets were compared to the rice reference genome using blast (version: 2.2.21; *e*-value < 1*e* − 20; [48]) and genomic coordinates were assigned to each contig based on the best blast hit. For multiple occurrences with identical hit-statistics both entries were retained (10% of contigs). Not located contigs were subjected to a comparison to the NT database of NCBI using blast (version: 2.2.21; *e*-value < 1*e* − 20; [48]).

### 2.7. Comparative Sequence Analysis

Genomic coordinates of the reference based and* de novo* assembly were combined using bedtools (version: 2.17.0; [35]) and* in house* developed Perl scripts. Visualizations of the read location in the genome were created using the Integrated Genome Viewer (IGV 2.3; [49, 50]).

Additional methylation datasets for rice from the publications of He et al. [51], Yan et al. [14], and Li et al. [52] were derived from the NCBI Sequence Read Archive (SRA, http://www.ncbi.nlm.nih.gov/sra/). The retrieved raw read information was subjected to the previously described preprocessing procedures and only reads with quality scores of more than Q30 and minimal length of 20 bp were used for further analysis. The reads were mapped with the previous described procedure to the rice genome.

The* de novo* assembled contigs of the saffron crocus dataset were compared to the protein sequences or rice, maize (5b.60; [39]), and* Brachypodium* (MIPS 1.2; [41]) using blast (version: 2.2.21; *e*-value < 1*e* − 10; [48]).

## 3. Results and Discussion

### 3.1. Enzyme Selection

**Figure 1:**
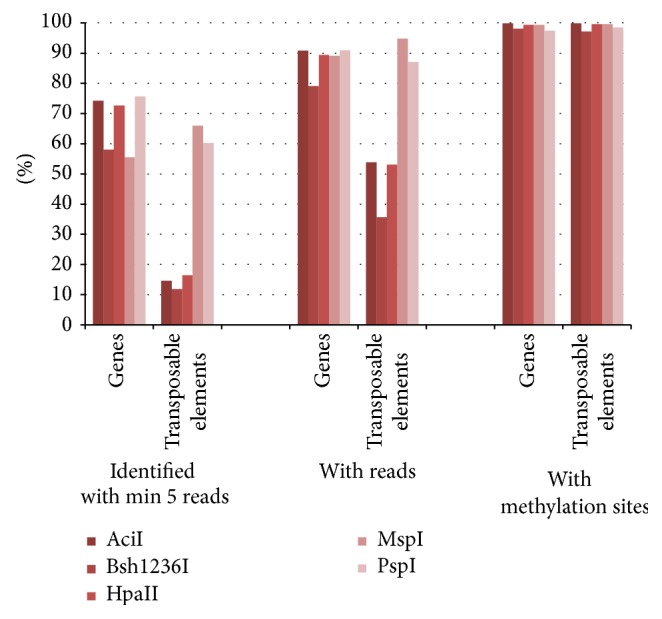
Genes and transposable elements identified in the rice genome with the methyl filtration technique. The regions comprised of at least five reads (left), and all regions (middle) show a clear depletion of transposable elements for AciI, Bsh1236I, and HpaII. On the right a representation of genes and transposable elements is given showing potential methylation sites within their gene space. All values are shown in percent based on the annotated 39.954 genes and 15.847 transposable elements.

In a first analysis five different methylation-sensitive enzymes were analysed for their enrichment potency. AciI, HpaII, and Bsh1236I are sensitive to CpG methylation, while MspI and PspGI are sensitive to CpH/WpG methylation. Although the restriction sites of the enzymes differ (AciI (CCGC), HpaII (CCGG), Bsh1236I (CGCG), MspI (CCGG), and PspGI (CCWGG)), they are present in almost every gene and transposable element allowing a genome wide study (Figure 1). For all enzymes the resulting fragments were isolated and sequenced and the obtained reads were mapped to the genomic sequence of rice. Considering all reads the resulting hypomethylated regions identified about 90% of the annotated gene models of rice for all five enzymes. A clear depletion of transposable elements was observed for the three CpG methylation-sensitive enzymes AciI, Bsh1236I, and HpaII whereas both CpH/WpG methylation-sensitive enzymes (MspI and PspGI) identified around 90% of the annotated transposable elements, therefore showing almost no depletion. Furthermore, the covered area of the identified transposable elements for the three CpG methylation-sensitive enzymes is very low in comparison to the covered area of the gene models. The depletion of transposable elements is even stronger when only hypomethylated regions comprising at least five reads are considered. This restriction causes a depletion of identified transposable elements to 14% and at the same time on average 68% of the gene models were identified. For HpaII and AciI even 74% of the annotated gene models were identified.

Based on these results HpaII and AciI were selected as enzymes showing the best gene space coverage and the highest transposable element (TE) depletion.

### 3.2. Isolated Hypomethylated Fragments Are Preferentially Located in the Gene Space

**Figure 2:**
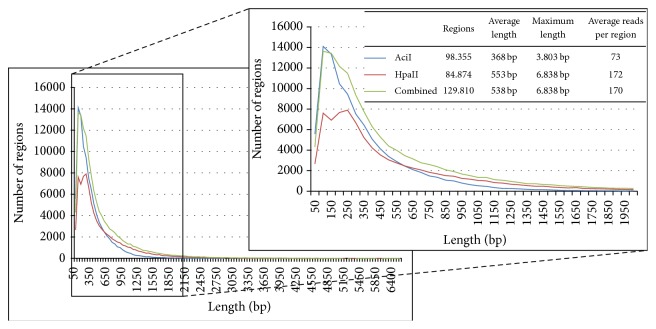
Length distribution of genomic regions identified for AciI, HpaII, and the combined dataset in rice. The length distribution of hypomethylated regions identified with the three datasets up to the maximal length is shown as well as a closer view to the region between 0 and 2.000 bp, where an increase in length is visible for the combined dataset. Additionally, the amount of regions, the average and maximum length, and the average reads per region are given.

**Figure 3:**
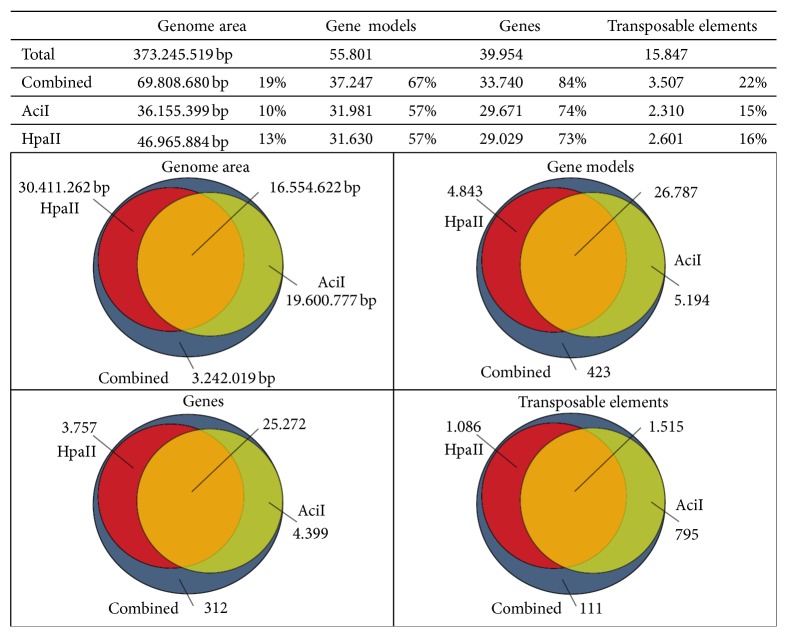
Overlap between the datasets in rice. The hypomethylated regions identified with the three datasets are compared focussing on genomic area, annotated gene models (including both genes and TEs and their surrounding +/− 1.000 bp regions), and genes and TEs separately. The total in each category is given in the table above while the overlap is visualized in the separate Venn diagrams.

The sequenced MF fragments were identified in the rice genome by mapping the high quality sequence reads to the genome sequence. The analysis was performed with a combination of both enzyme datasets, further referred to as combined dataset, and each dataset separately to investigate the complementarity of the two enzymes, AciI and HpaII. The combined dataset resulted in 129.810 regions representing 19% of the complete rice genomic sequence with an average coverage per bp of 61x integrating 80% of the reads. The single enzyme datasets identified 98.355 regions for AciI (coverage: 20x, reads: 69%) and 84.874 for HpaII (coverage: 40x, reads: 86%) representing 10% and 13% of the genome, respectively (see Figure 2 and Figure 3).

**Figure 4:**
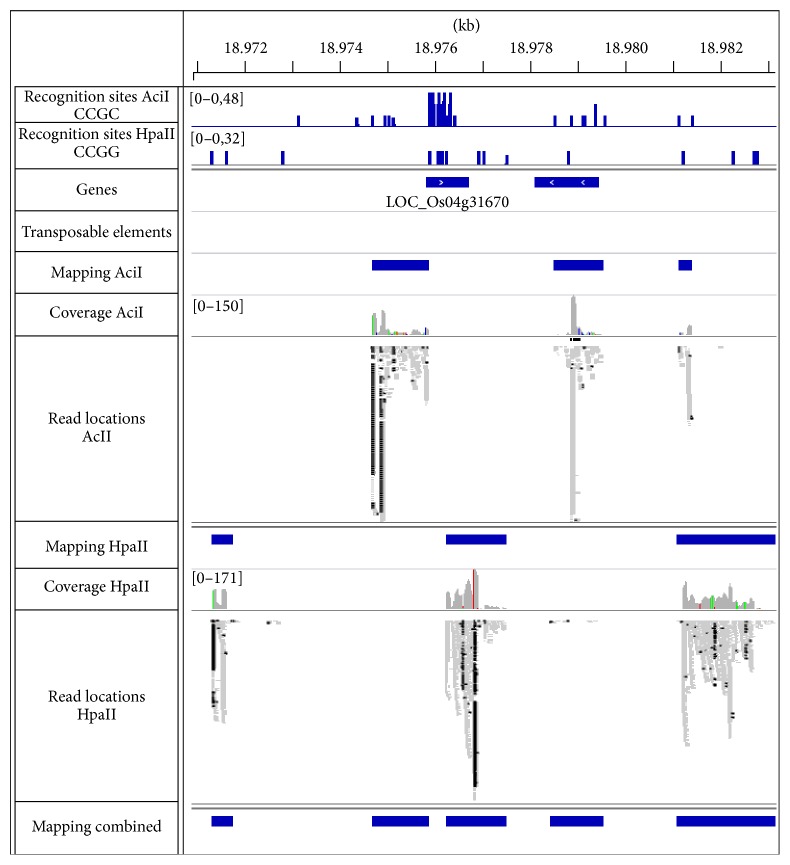
Complementary identification of genomic regions in rice due to restriction site locations. A detailed representation of the mapping results is shown for both enzymes, AciI and HpaII. The identified regions around the displayed gene differ due to the lack of recognition sites for the other enzyme. On the right, an example for overlapping but expanded regions is given.

Figure 3 shows the overlap between the three datasets. All regions identified with the datasets of the single enzymes were also identified with the combined dataset. The comparison between both enzyme specific datasets shows the complementarity of both enzymes in respect of the identified genomic area. Whereas an overlap between both enzymes is clearly present, the majority of the identified genomic area is identified by only one of the enzyme datasets. This difference is mainly caused by the genomic location of the specific restriction sites of the used enzymes.  Figure 4 depicts an example of this scenario where distinct and overlapping regions for both enzymes were identified due to absence of the recognition sites for the other enzyme. The locations of the specific sites also affect the length of the regions, which is also reflected by their increased length in the combined dataset compared to the single datasets, especially in the range between 200 and 1.000 bp (Figure 2), showing an advantage of using the combination of both enzymes. In addition, the combined dataset identified extra 3 Mb of the genome which was not detected with only one dataset. These additional regions contain reads from both single enzyme datasets but did not exceed the minimal coverage applied as quality insurance within each individually. Therefore they represent overlapping regions between both single enzyme datasets and could most likely also be identified separately by increasing the initial sequencing coverage per enzyme dataset.

**Figure 5:**
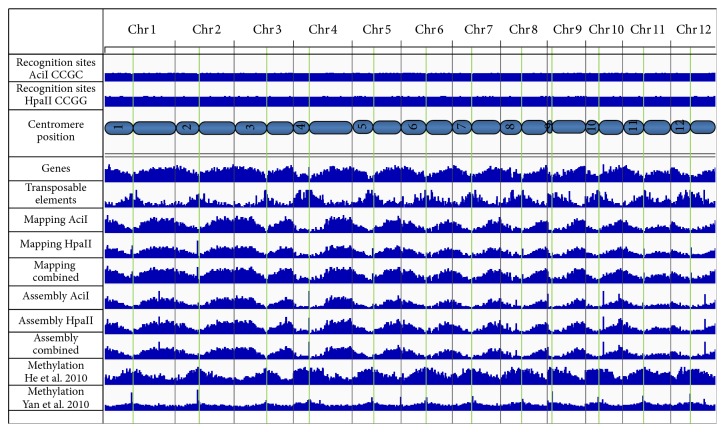
Genomic overview of hypomethylated regions in rice. The results of the* de novo* assembly and mapping approach are displayed for all three datasets (AciI, HpaII, and combined). In the upper panel the positions of the recognition sites are shown. The locations of annotated genes and TEs are depicted as well as the regions identified with the data of previous methylation studies (lower two tracks). The positions of the centromeric regions are also indicated and represented by green lines.

Analysing the location of the regions within the genome, it was found that 67% of the regions in the combined dataset were identified either within a gene model (both genes and TEs) or in their flanking 1.000 bp area. Separating genes and TEs clearly shows the depletion of TEs and the enrichment for the gene space (gene body and flanking 1.000 bp; see Figure 3). Of the 39.954 annotated genes 84% were identified with the combined dataset, whereas of the annotated 15.847 TEs only 22% showed a hypomethylated region. This observation is also clear in both single datasets (74% genes and 15% TE for AciI, and 73% genes and 16% TE for HpaII; see Figure 3), which is also reflected in the distribution of the isolated fragments across the genome corresponding clearly with the locations of the genes and opposing the distribution of TEs (Figure 5).

Detailed analysis of the locations emphasized the preference for the gene space and especially the gene body. In the combined dataset 75% of the regions were located within the gene space. Of those 67% are either located within or overlapping exons. This preference is even more prominent regarding the genomic area. 78% of the genomic area identified with the combined dataset is annotated as gene space and thereof 76% is associated with exons (see Table 1). Besides the enrichment for exons, another 16% of the isolated regions that are located in the gene space represent parts of the upstream 1.000 bp regions, of which 57% show known transcription factor binding sites.

Further comparative analysis with previously published results showed that 20% of the identified upstream area corresponds to regions of open chromatin in rice seedlings [53] indicating a potential active state of promoter elements in gene regulation. The isolation of hypomethylated regions via MF does therefore not only provide a representation of coding sequences within the gene space but does also provide potentially active regulatory upstream regions.

### 3.3. Sequencing Coverage Simulation to Identify the Hypomethylome of the Whole Genome

**Figure 6:**
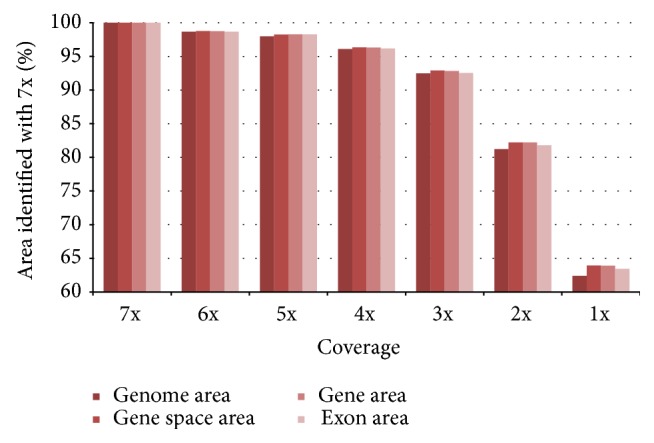
A simulation performed in rice to estimate the minimal coverage necessary to identify the hypomethylome of the whole genome was performed by randomly selecting reads from the combined dataset with 7x coverage to represent different coverage thresholds. The reads were allocated to the genome sequence and compared to the result of the complete dataset (100%) regarding genome area, gene space area, gene area, and exon area.

The overall sequence coverage was estimated based on the size of the complete genome sequence as 7x for the combined dataset, 3x for AciI, and 5x for HpaII, respectively, providing a reliable base for the study of the hypomethylated regions of the complete genome. For estimating the minimal coverage necessary to identify the hypomethylome of the whole genome, a simulation was performed by randomly selecting reads from the complete dataset to represent different coverage thresholds (2–7x) and remapping these to the genome sequence. The recovery of the allocated regions of the simulation datasets with the complete dataset was calculated together with the identified genomic area and the location within the gene space, gene body, and exon area, respectively. The data showed only a slight decrease in identified genomic area for 6x and 5x coverage. Also using a coverage of 4x results in 96% of the identified genomic area. But a clear decrease to 81% compared to the complete dataset can be observed with a coverage of 2x, while with a coverage of 3x still 92% were identified (Figure 6). The decrease of gene space, gene body, and exon area showed a very similar distribution.

Therefore we recommend a minimal coverage of 3-4x for similar studies. Hence, both single enzyme datasets also show enough coverage to represent the hypomethylome of the whole rice genome.

### 3.4. *De Novo* Assembled Contigs of Hypomethylated Regions Are Highly Similar to the Results of the Reference Based Identification

The* de novo* assembly of the datasets resulted in 187.168 contigs for the combined dataset integrating 82% of the reads, 129.402 contigs for the enzyme AciI (reads: 70%), and 111.390 contigs for HpaII (reads: 87%). The assembled contigs were located in the rice genome, where 95% could be identified. The contigs which could not be located within the genomic sequence consist mainly of the additional reads (2%) that could be assembled but could not be located with the mapping approach. A similarity search to the NT database of NCBI indicated no genic origin for these contigs suggesting a nongenic origin of the respective fragment with lower evolutionary selection pressure. Hence these regions might show more differences to the reference genome and could therefore not be identified based on similarity thresholds.

The genomic area represented by the* de novo* assembled contigs overlapped to 95% with the genomic area identified by the mapping approach. One fifth of the nonoverlapping genomic area was gained by the slightly longer regions produced by the assembly approach. However, the majority (about 80%) is mainly located in distinct regions close to the centromere or genomic chloroplast and mitochondrial regions as depicted in Figure 5. The* de novo* assembled contigs which could be located in these genomic chloroplast and mitochondrial regions showed differences to the plastid genomes. Therefore these contigs most likely do represent genomic hypomethylated regions, especially as all reads which showed high similarity to the plastid sequences of the chloroplast and the mitochondrion were filtered in the initial preprocessing. However they show also enough sequence differences to the published genomic sequence to prevent their identification with the mapping approach, indicating sequence variations between individuals which could hint to regions under lower selective pressure.

However these differences originate mainly from the HpaII dataset. The enzymes HpaII and AciI cleave only at a potential cut site if nonmethylated cytosines are present, therefore enriching hypomethylated regions. The observed difference in the two datasets would suggest the differential methylation of the recognition sites of the two enzymes, since these regions show evenly distributed recognition sites for both enzymes.

Comparing the hypomethylated regions identified in our system with the data of Yan et al. [14] representing methylated regions obtained by immunoprecipitation, we found 3% overlap. This overlap is mainly located in the regions identified only by HpaII. This contradicting information also suggests a different kind of methylation in these regions which does not prevent HpaII to cleave at its recognition site, while still preventing cleavage by AciI. The observed small overlap between the hypomethylated regions and methylated areas is also confirmed in the comparison to other datasets (Figure 5). A comparison of our hypomethylated regions with the methylation study of He et al. [51] indicated methylation in 8% of the genomic area identified as hypomethylated in our study. Those regions might represent areas which are differentially methylated in different individuals, developmental stages, or tissues. Similar results have recently also been shown in maize [54].

Our results of 19% identified hypomethylated regions in the rice genome are furthermore in good agreement with data of a previous study stating 76–91% genome coverage of methylated regions [52].

### 3.5. Applicability of the Method to Other Genomes

The nearly identical results of both the reference based mapping and the* de novo* assembly demonstrate that a reliable representation of the hypomethylated regions in a genome can be identified not only if a reference genome sequence is available but also by applying a* de novo* assembly approach. The applicability of the method is however dependent on the frequency of the recognition sites of the applied enzymes, which is in rice 6.2 sites/kb for AciI and 2.8 sites/kb for HpaII. An* in silico* analysis of several fully sequenced genomes showed a similar frequency in monocotyledon plants (AciI: maize 5.7 sites/kb, sorghum 4.1 sites/kb,* Brachypodium* 6.5 sites/kb; HpaII: maize 3.3 sites/kb, sorghum 2.2 sites/kb,* Brachypodium* 3.5 sites/kb). In other angiosperms and gymnosperms the frequencies are less but similar to frequencies observed in the human genome, where these enzymes are also used to study genome wide methylation patterns [21] (AciI: human 1.1 sites/kb,* Arabidopsis* 1.7 sites/kb, poplar 1.0 sites/kb, grapevine 0.9 sites/kb, Norway spruce 0.9 sites/kb; HpaII: human 0.8 sites/kb,* Arabidopsis* 1.1 sites/kb, poplar 0.7 sites/kb, grapevine 0.7 sites/kb, Norway spruce 0.5 sites/kb). This renders the presented technique highly applicable for nonmodel organisms where no genome sequence is available.

### 3.6. Applicability to the Large Genome of Norway Spruce

Despite the advances in sequencing technologies and the still increasing amount of sequenced genomes in the last decade one challenging issue remains especially for large plant genomes: their high amount of repetitive regions in the genome sequences. Genome sizes in plants range from 63 Mb up to about 150 Gb [55–57]. While gene size and number are rather constant with 30.000–50.000 genes, the differences in genome size are mainly due to the abundance of repetitive DNA which represents the majority of the genomic sequence [33, 36, 58, 59]. For example, the 20 Gb nuclear genome of Norway spruce contains 70% high copy number repeats and only about 2.4% of the nuclear genomic sequence, characterized as genes or gene-like fragments (possible pseudogenes) [33]. Repetitive regions have proven to be the main challenge in genome assembly approaches especially when using whole genome shotgun approaches. It has been suggested that silencing instead of chromosome rearrangement is the predominant mechanism in Norway spruce to deal with the high amount of repetitive regions [33]. One of the primary mechanisms that cause gene silencing is the methylation of DNA [4, 16], which renders Norway spruce a good example to emphasize the applicability of our technique to large genomes. Our technique was applied to isolate hypomethylated fragments from different samples of Norway spruce using only HpaII. Additionally non-filtrated genomic DNA was sequenced as comparison. Each dataset was treated and mapped to the published genomic sequence as described for the rice datasets. The resulting sequence reads represent an average coverage of the Norway spruce genome of 0.2x in the four samples and about 1x for the random genomic sequences.

**Figure 7:**
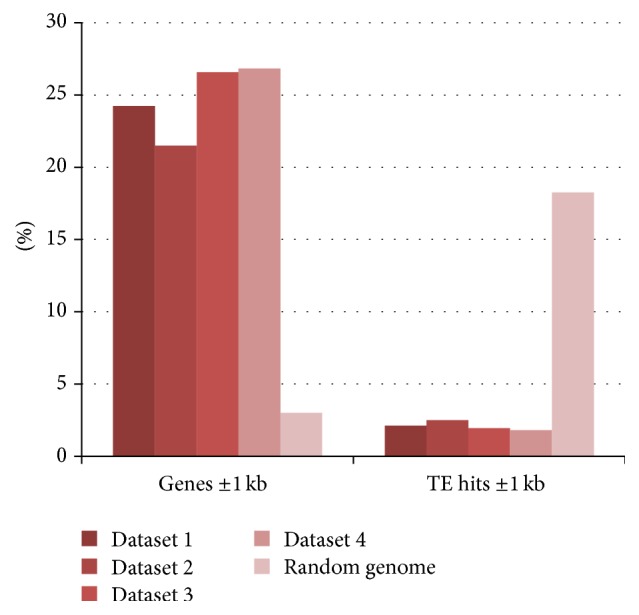
Reads located within the gene space (gene and surrounding +/− 1.000 bp regions) and the annotated TE (including surrounding +/− 1.000 bp regions) in Norway spruce. A clear enrichment of reads derived from gene regions and a clear depletion of reads derived from TE regions are shown.

Due to the lower coverage in these datasets we do not expect to gain the complete hypomethylome, although we observe the same advantages of depletion of transposable elements and the enrichment for the gene space. Particularly the comparison to the non-filtrated genomic dataset highlights both effects of our technique (Figure 7). This clear depletion and the similarity of the results to the rice results show that our approach is applicable also for the large Norway spruce genome and is not affected by the different composition of the genome or other repeat-classes as it depends on the DNA methylation pattern in the genome. However, as has been seen in the rice data, different methylation pattern can influence the detection. These results show the applicability of the technique to large genomes.

### 3.7. Applicability to Polyploid Genomes

Additionally, our technique has been applied to the triploid saffron crocus genome using only HpaII on one sample. The resulting reads have been preprocessed and* de novo* assembled as described for the rice datasets. The genome of saffron crocus has so far not been sequenced mainly due to its complexity. Its genome was estimated to have a size of 10.3 Gb [60]. In addition to its size it is a triploid genome most likely derived through crossing between two closely related species [60–62], introducing the complexity of polyploidy and different allelic variants to the analysis which could affect the quality and reliability of the assembly and the gene space detection. However, we did not observe a decreased quality of the* de novo* assembly as the analysis resulted in 13.986 contigs integrating 81% of the sequence reads. Sequence comparison to other monocotyledon plants showed a similar depletion of transposable elements (1%) and a similar enrichment for genes as 26% of the assembled contigs show similarity to protein coding genes. The identification of regions located in the flanking regions is not directly possible as no reference genome is available; however, 47% of the contigs show known regulatory elements, indicating the isolation of active regulatory regions. These results indicate that with our approach the detection of the gene space including regulatory regions is not affected when applied to polyploid genomes.

Although the overall genome coverage is rather small (0.2x), the obtained results are comparable to the data of Norway spruce and rice since a similar enrichment for the gene space and at the same time a clear depletion of transposable elements was detected. The data obtained on saffron crocus gave further support for the universal applicability of the presented method on a wide range of plant genomes, including also complex polyploid genomes.

## 4. Conclusions

Because of the large size and high complexity of many plant genomes, particularly those of important crops, gene-enriched sequencing strategies have been designed as an alternative to whole genome sequencing in an attempt to capture the gene space (genes plus regulatory elements) of such genomes. One of these enrichment techniques, called methyl filtration (MF), takes advantage of the difference in methylation state of cytosine residues being present between the gene space and repetitive elements.

With the present study performed on the model organism rice we demonstrate that with an improved MRE-seq method followed by* de novo* assembly, the hypomethylome and thus the gene space of a plant can be easily accessed. Using two methylation-sensitive restriction enzymes (HpaII and AciI), 84% of the annotated coding regions of the rice genome could be isolated, meanwhile, reducing the amount of isolated transposable elements to one fifth. The latter is of utmost importance to enrich the gene space as most plant genomes consist of 50–80% of repetitive elements including TEs. The presented method filters the genes including exons and introns as well as their up- and downstream flanking regions where regulatory elements are located. This represents a clear advantage over traditional transcriptome analysis approaches, which provide sequence data only of exons of active genes.

*De novo* assembly shows almost identical results as reference based mapping of the sequence reads, demonstrating the applicability of the MRE-seq approach to nonmodel plant species where no fully sequenced genome is available. The coverage needed for generating an informative snapshot of a given genome is estimated as 3-4x. Small scale studies in the large genome of Norway spruce and the polyploid saffron crocus demonstrate the depletion of transposable elements and enrichment for the gene space in nonmodel species and complex plant genomes.

The overlap of our results with methylation data from previous studies confirms the high potential of MRE-seq for being applied in a wide range of scenarios for the direct analysis of methylation differences, for example, between ecotypes and individuals, and within and across species. This new and easy technique allows the fast and inexpensive generation of data necessary for studying the role of epigenetics as a source of adaptive variation in natural populations as well as crop plants. It is especially helpful with regard to studying and understanding the genomes of nonmodel organisms.

## Figures and Tables

**Table 1:** Allocation of the identified hypomethylated regions to the gene space in rice: the position of the identified hypomethylated regions is given in respect of the gene space together with the genomic area.

	AciI		HpaII		Combined	
*Hypomethylated regions *						
Total	98.355		84.874		129.810	
Gene space (±1.000 bp)	76.295	78%	62.655	74%	96.717	75%
Upstream 1.000 bp	13.342	17%	10.876	17%	15.629	16%
Downstream 1.000 bp	7.739	10%	6.398	10%	9.556	10%
Gene body	55.214	72%	45.381	72%	71.532	74%
Exon	48.933	64%	41.034	65%	64.622	67%
Intron	6.281	8%	4.347	7%	6.910	7%

*Genomic area [bp] *						
Total	36.155.399		46.965.884		69.808.680	
Gene space (±1.000 bp)	28.580.311	79%	36.521.506	78%	54.732.127	78%
Upstream 1.000 bp	4.439.728	16%	5.111.923	14%	6.931.498	13%
Downstream 1.000 bp	2.650.795	9%	2.949.893	8%	4.235.613	8%
Gene body	21.489.788	75%	28.459.690	78%	43.565.016	80%
Exon	19.739.225	69%	27.027.474	74%	41.407.771	76%
Intron	1.750.563	6%	1.432.216	4%	2.157.245	4%
